# Vacant Professorships

**Published:** 1849-05

**Authors:** 


*Vacant Professorships.*—Several professorial chairs in different Medical Institutions have lately been made vacant by deaths and resignations. The demise of the late lamented Drs. Carpenter and Harrison, leaves two vacancies in the Louisiana Medical College. Dr. Thomas D. Mitchell has resigned the chair of Materia Medica in Transylvania University, Lexington Kentucky, and Dr. Henry M. Bullett, of Louisville, has been appointed to fill his place. Dr. Donne, has retired from the chair of Anatomy in the Memphis Medical College. Dr. Bartlett, of Transylvania University, has resigned the chair of Medicine. Dr. Drake has severed his connection with the Louisville Medical School, and has resumed the practice of Medicine in Cincinnati, Ohio. Dr. Fitch, late Professor of Theory and practice of Medicine in the Rush Medical College, Chicago, has resigned. None of the foregoing vacancies are filled, to our knowledge, with exception of the chair of Materia Medica at Transylvania, as already stated. Here are chances for aspirants for professorial labors!

